# An account of a pregnancy-related fracture resulting in lower extremities deep vein thrombosis and inferior vena cava compression: A case report

**DOI:** 10.1097/MD.0000000000044846

**Published:** 2025-10-10

**Authors:** Qianqian Jiang, Yu Huang, Tianyi Zhu, Chong Li, Jiatao Li, Yaqi Wang, Ruijiao Gao, Liang Li, Xiangdong Liu, Yanrong Zhang, Yunsong Li

**Affiliations:** a Department of Vascular Surgery, The Third Hospital of Hebei Medical University, Shijiazhuang, P.R. China; b Department of Laboratory, The Third Hospital of Hebei Medical University, Shijiazhuang, P.R.China.

**Keywords:** case report, IVC compression, lower extremity fracture, pregnancy, VTE

## Abstract

**Rationale:**

Venous thromboembolism (VTE) is a dangerous condition that has a major global impact on morbidity and death. VTE risk factors include both pregnancy and fracture.

**Patient concerns:**

The patient was hospitalized during pregnancy due to a right lower extremity fracture that was complicated by deep vein thrombosis (DVT) – a condition carrying a risk of fatal pulmonary embolism – and was found to have inferior vena cava (IVC) syndrome.

**Diagnoses:**

DVT of the right lower extremity, right femoral fracture, pregnancy, and IVC syndrome.

**Interventions:**

The patient underwent several procedures, including IVC filter placement, fracture surgery, cesarean section, and thrombus aspiration, in addition to receiving anticoagulation therapy and other medications.

**Outcomes:**

After treatment, the patient’s right lower extremity swelling subsided, with no symptoms such as chest tightness or chest pain. Both lower extremities were fully mobile, and the patient was discharged without complications. Her infant was healthy.

**Lessons:**

Although no pregnancy-related VTE preventive guidelines have specified IVC compression as an evaluation item, we took into account the patient’s right femoral fracture and IVC compression, which were linked to the rapid onset of DVT in this case. More research is necessary to determine whether IVC compression and pregnancy-related VTE are clearly correlated.

## 1. Introduction

Globally, venous thromboembolism (VTE), which includes pulmonary embolism (PE) and deep vein thrombosis (DVT), is a dangerous condition with high morbidity and fatality rates.^[1]^Braking, being overweight, and severe fractures are common risk factors for VTE. Pregnant women are 4 times more likely to develop VTE than nonpregnant women, making pregnancy a significant risk factor for the condition.^[2,3]^ To make sure that the right risk reduction strategies are put in place, it is crucial to be knowledgeable about the risk factors for pregnancy-associated VTE. High-quality data support on prevention, therapy, and other related topics is still lacking, though.^[4]^ A case of right-sided DVT in a pregnant patient who suffered a lower extremity fracture is described in this article. In addition to summarizing and discussing the risk factors of pregnancy-related venous thromboembolism (PA-VTE), this case illustrates the diagnostic and therapeutic problems of this complex scenario. The goal is to offer fresh perspectives on the PA-VTE risk assessment.

## 2. Case presentation

The patient was a 37-year-old woman, G5 P1, 35 weeks pregnant, with right thigh edema, pain in the lower right leg, and a fractured right femoral neck from a fall 13 hours before admission. Due to “two weeks of umbilical cord wrapping,” she had previously undergone a lower uterine segment cesarean section at 39 weeks of pregnancy. The fetal heart rate monitor displayed an NST response, and the obstetric ultrasound revealed “intrauterine pregnancy with a single live fetus in cephalic position and 1 week of umbilical cord wrapping” in the current pregnancy. The patient’s self-monitored fasting glucose was 5. 5–5. 7 mmol/L, and during this pregnancy, her oral glucose tolerance test was 5. 57–13. 09–10. 96 mmol/L. Because fracture surgery was desired, a cesarean section was scheduled at the 35th week of pregnancy. The patient had no prior history of PE or DVT in the lower extremities.

**Figure 1.**
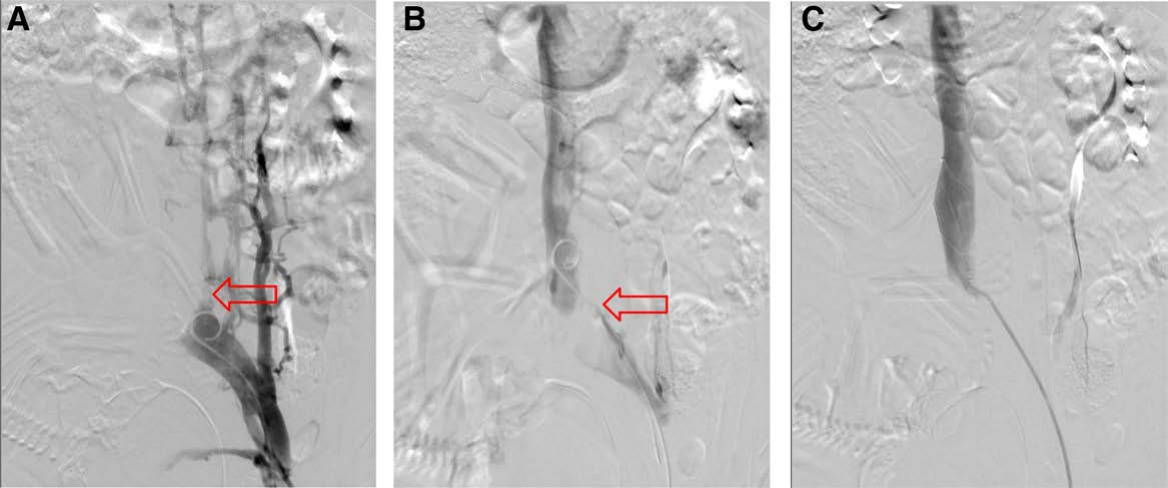
When DVT is initially discovered, inferior vena cava angiography demonstrates compression of the inferior vena cava. (A) Visible fetal compression of the IVC. (B) Blood flow in the proximal segment of the IVC is unobstructed, while the distal segment of the IVC is completely occluded. (C) Placement of a IVCF in the IVC segment below the renal vein opening. DVT = deep vein thrombosis, IVC = inferior vena cava.

The distal part of the right common femoral vein extended to the upper segment of the deep femoral vein thrombus, according to the lower limbs’ deep vein ultrasound obtained after admission. Despite the absence of symptoms like dyspnea, the patient’s right thigh was noticeably enlarged. PE, which can embolize the pulmonary vascular system if treatment is not received, is more common in patients with proximal DVT than distal DVT, and it poses a serious risk to the mother and fetus.^[1]^ On the same day, inferior vena cava (IVC) filter (Visee) insertion was done to avoid a potentially catastrophic PE. According to intraoperative imaging, the left common iliac vein had smooth blood flow, the vein’s opening was noticeably thicker, the patient’s IVC was not visible, and there were numerous collateral formations surrounding it. Additionally, radiography clearly showed fetal compression of the patient’s IVC (Fig. 1A). The blood flow in the renal vein’s IVC was open when the tail catheter was applied through the little opening. The IVC showed no signs of stenosis or vena caval thrombosis (Fig. 1B). progressively the guidewire’s IVC filter. Gently pull the sheath back once the wire is in position, then slowly insert the IVC filter into the infrarenal IVC (Fig. 1C).

At the mother’s and her family’s desire, a lower uterine segment cesarean section was carried out under epidural anesthesia on the day of admission. Vital signs for both the mother and the fetus were steady, and the procedure proceeded without incident. There is a significant chance of bleeding because of the patient’s recent surgical trauma. As a result, after surgery, keep giving low-molecular-weight heparin (LMWH) 5000 IU q12. The procedure proceeded without incident 9 days following the cesarean section. It involved iliac bone removal, closed reduction hollow nail internal fixation of the right femoral neck fracture, and femoral neck bone grafting under general anesthesia and nerve block. She continued to provide LMWH 5000IU q12 anticoagulant therapy following the procedure.

**Figure 2.**
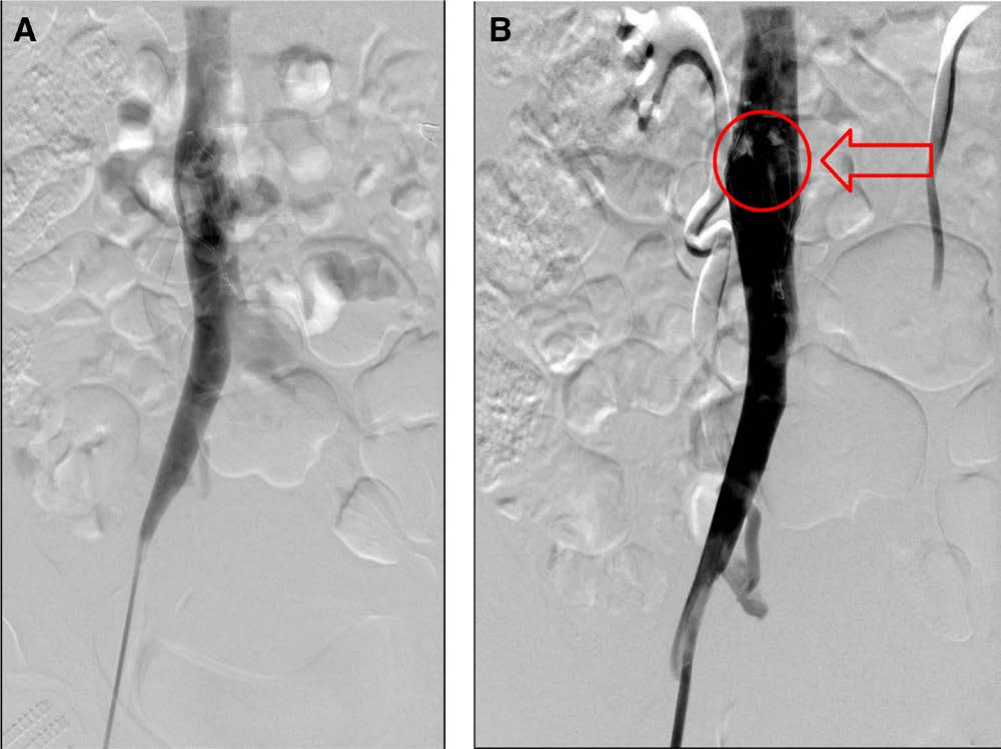
After the patient’s cesarean section, IVC angiography revealed filter-related thrombosis. (A) Blood clots are caught by the IVCF. (B) There is still additional thrombus evident at the proximal end of the IVCF following the thrombus aspiration technique. IVC = inferior vena cava.

Ten days after cesarean section, the deep vein color ultrasound of the lower limbs was reexamined, which showed that the blood flow in the right common femoral vein was unobstructed, with no obvious signs of thrombosis in the right fibular vein and the right calf. The results of a follow-up deep vein ultrasound examination of the lower limbs revealed that the right peroneal vein and the right calf intermuscular vein thrombosis were both free of thrombosis. The following possibilities are taken into consideration: a. The patient’s thrombus load was considerably decreased with anticoagulation; b. The patient’s thrombus load rose, but the proximal thrombus moved into the IVC filter. Fourteen days after the cesarean section, a right lower extremity venography was conducted. With no noticeable filling defects, no substantial IVC compression images, and more filling defects in the IVC filter given that the filter was obstructing thrombus (Fig. 2A), blood flow in the right complete femoral vein was unhindered. After thrombus aspiration, reimaging revealed smooth blood flow in the filter, the right iliac vein, and the IVC. However, more filling flaws were still seen at the proximal end of the filter (Fig. 2B), therefore it was decided to replace the filter (Biovas).

**Figure 3.**
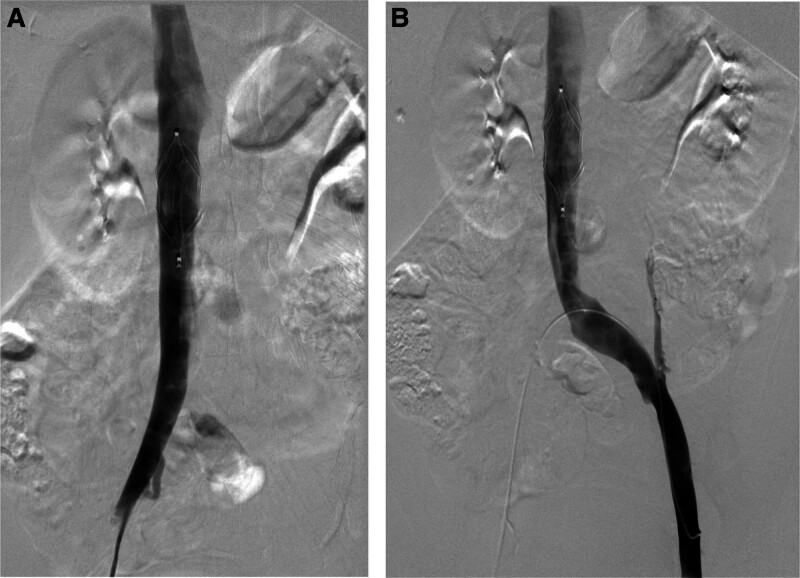
The IVC angiography revealed smooth blood flow following 3 weeks of anticoagulation. (A) The right common femoral vein, iliac vein, and IVC were flowing well without filling defects. (B) The left common femoral vein, left iliac vein, and IVC were flowing well and that the filters were removed. IVC = inferior vena cava.

She continued to use low-molecular heparin 5000IU q12 anticoagulant medication after being released from the hospital. 26 days after the cesarean section, D-Dimer 1.14 ug/ml was rechecked, and lower limb venography was done that same day. Figure 3A showed that the right common femoral vein, iliac vein, and IVC were flowing well without filling defects, while Figure 3B showed that the left common femoral vein, left iliac vein, and IVC were flowing well and that the filters were removed. Table 1 displays specifics of the patients’ anticoagulant treatment and D-dimer levels while they were in the hospital. The anticoagulation regimen and D-dimer values during hospitalization are shown in Table 1.

**Table 1:** D-dimer and anticoagulation medication during hospitalization.

Time	Regimen of anticoagulation	D-dimer (μg/mL)
Julu 7, 2024	LMWH 5000U q12	27.99
July 11, 2024	LMWH 5000U q12	4.09
July 21, 2024	LMWH 5000U q12	4.17
August , 2024	LMWH 5000U q12	1.14

## 3. Discussion

### 3.1. Risk factors for PA-VTE

Pregnancy-related physiological changes, such as variations in the quantity of clotting factors and the pregnant uterus compressing the vasculature, raise the risk of VTE.^[5]^ The patient in this article had high risk factors for VTE, including mobility issues and a femur fracture. The patient developed an isolated DVT in the right lower limb’s proximal region. The patient’s pregnancy and fracture stimulation are thought to be connected to the lower leg DVT. According to Kilkenny K et al., roughly 70% to 90% of DVTs during pregnancy occur on the left side, 71% proximally, and 64% are restricted to the femoral and proximal iliac veins. About 30% to 50% of lower extremity DVTs are distal, and left-sided DVTs are slightly more common in the general population (58%).^[1]^But in this instance, the right deep vein had the majority of the DVT, which was thought to be connected to the patient’s fracture site. Ultrasonography showed thrombosis in the right femoral vein 13 hours after the fall. This indicates that there are strong prothrombotic factors present because the thrombus formed quite quickly. Furthermore, there was no damage or compression of the right femoral vein found during the preoperative ultrasound assessment, and there was not much of a delay between the fall and the ultrasound test. Thus, in conjunction with the intraoperative circumstances, we deduced that the primary cause of thrombosis was blood flow stasis brought on by IVC compression, in addition to fracture and pregnancy-induced hypercoagulability.

It is advised that all early pregnant women undergo routine risk assessments for PA-VTE using the Royal College of Obstetricians and Gynaecologists Risk Assessment Model (RCOG RAM) as soon as the pregnancy is confirmed and then at regular intervals of every 3 months. Additionally, a risk assessment for PA-VTE should be conducted if new risk factors or complications emerge, or if the patient is hospitalized for any reason, according to the recently published International Consensus Statement on the Prevention and Management of Venous Thromboembolism, 2024.^[6]^ Because of the presence of risk factors, including age (>35 years), obesity (BMI = 30), desire to have surgery for a fractured right femur during pregnancy, lower extremity braking (score of 6), and prolonged hospitalizations within the puerperium (≥3 days) following a fracture during pregnancy, thromboprophylaxis should be considered in this patient based on the RCOG RAM. We reassessed the patient’s RCOG RAM score prior to the pregnancy fracture, and it was 2, because the patient’s period between the lower extremity fracture and the DVT discovery was actually quite short – <24 hours. Thromboprophylaxis was actually not required.^[7,8]^ It was still thought to be related to the patient’s IVC compression because, upon reexamining the entire case, we discovered that the patient had a physiological change of uterine compression of the IVC during pregnancy and that the patient’s acute DVT was primarily concentrated in the right femoral vein. However, despite studies on IVC compression by ultrasound during pregnancy, no guidelines include IVC compression as a required screening test during pregnancy. This is in contrast to the numerous guidelines on risk assessment, treatment, and management of VTE during pregnancy, which do not include the degree of IVC compression/IVC diameter in the risk assessment table. Thus, in order to identify VTE early and avoid it, the author suggests that IVC compression be part of the required items throughout pregnancy and be part of the risk assessment of VTE during pregnancy.

### 3.2. Options for treatment in the complicated situation of pregnancy-related deep vein thrombosis in the lower limbs with fractures

The patient suffered lower extremity deep vein thrombosis, a fracture, and pregnancy all at the same time. In order to avoid intraoperative thrombus dislodgement that could result in a PE, a multidisciplinary debate led to the decision to perform a cesarean section and femoral fracture following IVC filter installation. reimaging revealed filter-related thrombosis 2 weeks later, but thrombosis of the right peroneal and right interosseous veins occurred, indicating that the patient’s thrombus had advanced, and the right common and deep femoral vein thrombus vanished, which was thought to be thrombus dislodgement. The 2 procedures were thought to have contributed to the patient’s thrombus dislodgement, which is a risk factor for thrombus dislodgement. Nevertheless, the patient’s thrombus worsened, which was thought to be caused by insufficient anticoagulation. Given that the patient was nursing and that the CHEST guidelines recommend a 3-month anticoagulation therapy for acute VTE patients without contraindications and that extended anticoagulation therapy should not be offered to VTE patients with primarily transient risk factors (pregnancy, surgery).^[6]^After being discharged, patients are required to give themselves LMWH subcutaneous injections. We advise patients to receive LMWH 5000 IU q12 therapy for a minimum of 3 months following discharge and to have frequent follow-up appointments for their convenience.

## 4. Conclusion

In this case report, we describe a pregnant patient who was diagnosed with DVT of the lower extremities following a fracture. Despite the fact that no PA-VTE prevention guidelines have included IVC compression as an evaluation item to date, and whether IVC compression is clearly related to PA-VTE has not been confirmed by high-quality studies, we believe that the patient’s right lower extremity DVT is related to the patient’s IVC compression, and the examination for IVC compression/IVC diameter should be included in the risk assessment list for RA-VTE.^[9,10]^ Pregnancy-related fracture and thrombosis diagnosis and treatment still face numerous obstacles, such as a lack of high-quality data and divergent expert opinions and recommendations. For the best care, interdisciplinary dialogue and collaboration are essential. In order to better comprehend this population and create more accurate and logical study protocols, we must place a strong priority on interdisciplinary collaboration and carry out prospective clinical management studies and high-quality trials.

## 5. Patient’s perspective

I shattered my right femur in an unintentional fall during my pregnancy. My right thigh swelled and felt sensitive to the touch shortly after. A vascular ultrasound performed after my hospital admission showed that I had a blood clot in my right femoral vein. The doctor suggested that I have a filter implantation procedure for safety. Angiography during the treatment showed that my baby was squeezing the IVC, which the doctor felt was the main cause of the blood clot’s quick creation. As expected, my right thigh’s swelling went away following the cesarean section, and I later had fracture surgery. But I was alarmed when thrombus development was found in the filter during a follow-up IVC angiography. I could have had PE symptoms if the filter implantation procedure hadn’t been done. I was also relieved that the IVC’s compression had been released and the thrombus had progressively settled.

## Acknowledgments

We have obtained the patient’s permission to publish the patient’s relevant data, including images, in the Journal of *Medicine*.

## Author contributions

**Conceptualization:** Qianqian Jiang, Yu Huang, Yanrong Zhang, Yunsong Li.

**Data curation:** Qianqian Jiang, Tianyi Zhu, Yunsong Li.

**Formal analysis:** Chong Li, Jiatao Li, Yaqi Wang.

**Funding acquisition:** Yunsong Li.

**Investigation:** Qianqian Jiang, Tianyi Zhu, Chong Li, Yaqi Wang, Ruijiao Gao.

**Writing – original draft:** Qianqian Jiang, Yu Huang, Jiatao Li, Liang Li, Xiangdong Liu, Yanrong Zhang, Yunsong Li.

**Writing – review & editing:** Qianqian Jiang, Yu Huang, Liang Li, Xiangdong Liu, Yanrong Zhang, Yunsong Li.
